# Predictive performance of population pharmacokinetic models of imatinib in chronic myeloid leukemia patients

**DOI:** 10.1007/s00280-024-04644-w

**Published:** 2024-03-05

**Authors:** Jaya Shree Dilli Batcha, Vikram Gota, Saikumar Matcha, Arun Prasath Raju, Mahadev Rao, Karthik S. Udupa, Surulivelrajan Mallayasamy

**Affiliations:** 1 Department of Pharmacy Practice, Manipal College of Pharmaceutical Sciences, Manipal Academy of Higher Education, Manipal, India; 2 Department of Clinical Pharmacology, ACTREC, Tata Memorial Centre, Mumbai, India; 3 Titus Family Department of Clinical Pharmacy, Alfred E. Mann School of Pharmacy and Pharmaceutical Sciences, University of Southern California, Los Angeles, USA; 4 Department of Medical Oncology, Manipal Academy of Higher Education, Kasturba Medical College, Manipal, India; 5 Center for Pharmacometrics, Manipal Academy of Higher Education, Manipal, India

**Keywords:** Population pharmacokinetic model, Pharmacometrics, Chronic myeloid leukemia, Imatinib, Dosing nomogram

## Abstract

**Background and aim:**

Chronic myeloid leukemia is a myeloproliferative neoplasm associated with the specific chromosomal translocation known as the Philadelphia chromosome. Imatinib is a potent BCR-ABL tyrosine kinase inhibitor, which is approved as the first line therapy for CML patients. There are various population pharmacokinetic studies available in the literature for this population. However, their use in other populations outside of their cohort for the model development has not been evaluated. This study was aimed to perform the predictive performance of the published population pharmacokinetic models for imatinib in CML population and propose a dosing nomogram.

**Methods:**

A systematic review was conducted through PubMed, and WoS databases to identify PopPK models. Clinical data collected in adult CML patients treated with imatinib was used for evaluation of these models. Various prediction-based metrics were used for assessing the bias and precision of PopPK models using individual predictions.

**Results:**

Eight imatinib PopPK model were selected for evaluating the model performance. A total of 145 plasma imatinib samples were collected from 43 adult patients diagnosed with CML and treated with imatinib. The PopPK model reported by Menon et al. had better performance than all other PopPK models.

**Conclusion:**

Menon et al. model was able to predict well for our clinical data where it had the relative mean prediction error percentage ≤ 20%, relative median absolute prediction error ≤ 30% and relative root mean square error close to zero. Based on this final model, we proposed a dosing nomogram for various weight groups, which could potentially help to maintain the trough concentrations in the therapeutic range.

**Supplementary Information:**

The online version contains supplementary material available at 10.1007/s00280-024-04644-w.

## Introduction

Chronic myeloid leukemia (CML) is a myeloproliferative neoplasm associated with the specific chromosomal translocation in chromosomes 9 and 22, which is known as the Philadelphia chromosome (Ph). Around 95% of the patients with CML are detected with Ph chromosome [1]. In the general community, the diagnosis of CML is found to be 1–2 instances per 100 000 population. Adults with newly diagnosed leukemia account for about 15% of all leukemia cases [2]. Imatinib mesylate (IM) belongs to the potent class of anticancer drugs known as BCR-ABL tyrosine kinase selective inhibitors. It was introduced in the year 2000 and thereafter, it has become the gold standard for treatment of CML. The death rate due to CML has come down from the range of 10–20% to 1–2% after the introduction of imatinib [2, 3].

In patients with CML, imatinib is given at a standard dose of 400 mg in chronic phase and 600 mg in accelerated and blast phase [4], orally once daily, which is well tolerated. It has been noted that imatinib has high intra-individual variability (IIV) and between subject variability (BSV) in the pharmacokinetic parameters [5–7]. Despite the excellent response imatinib produces, some CML patients show resistance to drug or therapy failure after a primary response. Variations in plasma trough concentration (C_trough_) of Imatinib could affect clinical responses in CML, and it is also related to the occurrence of prominent adverse events (AEs) such as edema or fluid retention, neutropenia, grade 3 myalgia and others [6, 8–12]. Standard doses are used for treating different phases of CML; however, attaining clinical outcome in all the treated patients is a challenge. Trough concentrations greater than 1 µg/mL have been shown to help achieve higher efficacy[13–15]. Few studies have shown that if the trough concentration are kept between 1and 1.5 µg/mL, there is a higher chance of therapeutic success without drug resistance and a lower chances of developing AEs [8, 16]. Population pharmacokinetics (PopPKs) is a branch of pharmacometrics that comes from quantitative clinical pharmacology, which explores the sources and correlates to variability in drug concentrations [17].

Pharmacometrics in precision medicine considers variability and recommends dosage regimen according to the sub-populations and has broad applications in clinical onco-therapeutics [18]. This study was aimed to evaluate the predictive performance of the published population pharmacokinetics model for Imatinib in CML and propose a dosing nomogram based on the model which had a better prediction ability.

## Methodology

### Systematic review for identification of PopPK models

A systematic review was conducted by two reviewers independently to identify relevant literatures. If there was any difference in opinion during this process, then a third reviewer was consulted to resolve the issue and come to an agreement. The systematic search was conducted by following PRISMA (Preferred Reporting Items for Systematic Reviews and Meta-analyses) criteria in the PubMed and Web of Science databases. The articles published till December 31, 2022, were included in the review. The keywords used in the databases were “Imatinib”, “Population Pharmacokinetic”, “PopPK”, “Chronic myeloid leukemia” and, “CML”. Imatinib population pharmacokinetics in CML patients published in English language were included in the review. Conference abstracts, editorials and book chapters were excluded.

### Data extraction and model evaluation

Details of study like title and author details, study design, treatment and drug quantification details and pharmacokinetic model related data which includes model structure, parameter values, BSV, RUV, and covariates were collected.

### PopPK models replication

All selected PopPK models were replicated using the structural model and typical parameters obtained from the published values of each candidate model. Empirical Bayesian estimate was used to derive individual-based predictions for imatinib plasma concentration for each time point.

### Clinical data for external evaluation of PopPK models

The clinical data for 49 subjects was collected in a prospective observational study from CML patients, who were on Imatinib therapy through routine therapeutic drug monitoring. This study was conducted in ACTREC, Mumbai a tertiary care oncology hospital after approval from institutional ethics committee. The following demographics and clinical patient information were collected: gender, age, body-weight, height, liver function test, blood cells count, concomitant medication, single nucleotide polymorphisms (SNPs) which included ABCB1, ABCG2, CYP3A4 genotypes, and imatinib treatment information (Dose, sampling time, plasma concentration, stage of CML).

### Drug quantification

A high-performance liquid chromatography technique developed by Peng et al. [1] was used to ascertain the plasma drug concentration. Erlotinib was used as the internal standard and the method was validated over a linear range of 100 to 10,000 ng/ml [19].

### External evaluation of PopPK model predictive ability

The predictive performance of the selected PopPK model was performed with the external dataset using the PUMAS AI package. Graphical representations of the results were performed with R version 4.2.1. The PUMAS package version 2.3.0 with Julia computing language was the tool used to run all the pharmacometric simulations [20].

### Model evaluation using prediction-based metrics

Relative mean prediction error percentage [rMPE (%)], relative median absolute prediction error percentage [rMAPE (%)] and relative root mean squared error (rRMSE) are the most commonly used metrics for evaluating model bias and precision [21]. In this study, we used rMPE (%) to predict the bias of the PopPK models. The rMPE (%) was calculated by comparing the individual predicted concentration (C_ipred_) estimated by using the parameters in the structural and stochastic models to the final estimates of each PopPK model to the observed concentration (C_obs_) for each subject. The following equation was used to determine rMPE (%), rMAPE (%) and rRMSE:

$${\text{rMPE }}\left( \% \right) = \frac{1}{n}\mathop \sum \limits_{i = 1}^n \left( {\frac{Cipred - Cobs}{{Cobs}}} \right){*}100$$

$${\text{rMAPE }}\left( \% \right) = Median of\left| {\frac{Cipred - Cobs}{{Cobs}}} \right|$$

$$rRMSE = \frac{1}{n}\sqrt {{\mathop \sum \limits_{i = 1}^n \left( {\frac{Cipred - Cobs}{{Cobs}}} \right)^2 }}$$

## Results

### Literature search and study selection

**Fig. 1:**
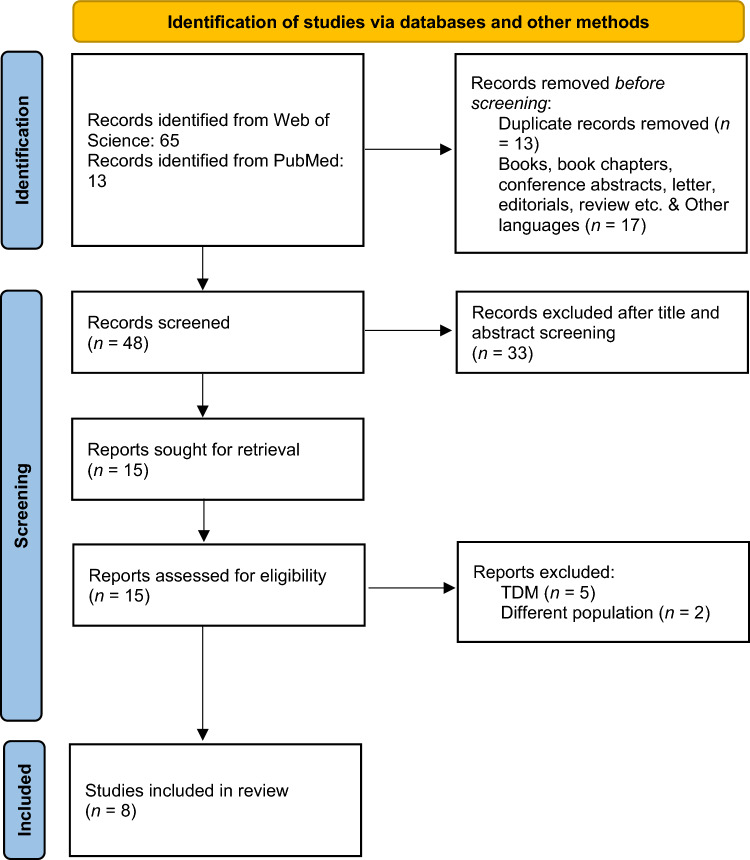
PRISMA flow chart

A total of 78 research publications were identified through the PubMed and Web of Science database searches. After the title and abstract screening, a total of 15 articles were left for review. Out of 15 articles, seven were excluded as they were therapeutic drug monitoring (TDM) studies and the PopPK model of Imatinib for gastrointestinal stromal tumors. Eight population pharmacokinetic studies of Imatinib in CML [22–29] met the inclusion criteria and were included in this study after the scrutiny of the literature. Supplementary Table 1 summarizes the demographic data of studies such as weight, dose, study population and covariates tested in the models. Supplementary Table 2 shows the search strategy and Fig. 1 depicts the study selection methodology. In most of the studies, the modeling tool used for the development of the PopPK models was NONMEM software package. The relation between different covariates and pharmacokinetic parameters and the values of BSV were reported in these studies. Visual predictive check and bootstrap analysis were the common reported approaches for model evaluation.

PK parameters such as Clearance (CL), absorption rate constant (Ka), and volume of distribution (Vd), IIV, RUV, and inferences from various studies are presented in Table 1. All the selected studies reported one-compartment model for imatinib with either first-order or zero-order absorption with or without lag time. Proportional error model was the most common error model used in these studies for explaining residual error.

**Table 1:** Study parameters and Inference

Author, year	Parameters						Inference
	CL (%RSE)	Ka (%RSE)	Vd (%RSE)	D1 (%RSE)	IIV (%)	RUV	
Ansari et al. 2016	11.8 (11.9)		349 (7.3)		CL–37.6 Vd–53.9	PE–27.2	The use of demographic covariate like weight has an influence on pharmacokinetics parameters
Golabchifar et al. 2014	10.8 (4)		278.3 (8)	1.384 (7)	CL–30.2 Vd–53.5 D1–36	PE–28.4	TDM of plasma Cmin is suggested at-least in patients who do not respond optimally to the treatment as there is a correlation between trough levels and molecular response
Gota et al. 2014	17.3 (3.7)		430 (9.7)	3.1 (14.2)	CL–37.6 Vd–0.73	PE–29.2 AE–82.8 (ng/mL)	Female patients had 15.2% lower CL than males and CL reduced by 23% from 40–80 years of age
Menon et al. 2008	10.8 (7.5)		284 (6.5)	1.67 (0.5)	CL–31.5 D1–92.6	PE–40.8	Among various absorption model, a zero-order process best explained the absorption of imatinib following oral administration
Schmidli et al. 2004	13.8 (67.8)		252 (58.2)	1.50 (fixed)	CL–31.9 Vd–31.4	PE–26 AE–0.062 (mg/L)	Doubling in weight and Hb values increased the imatinib CL
Wang et al. 2018	9.25 (4)	0.329 (36)	222 (14)		CL–42.6	PE–63.7 AE–3.137 (ng/mL)	Bodyweight has impact on imatinib pharmacokinetics
Widmer et al. 2006	14.3 (7.1)	0.61 (30)	347 (17 .9)		CL–36 Vd–63	PE–31	Doubling in bodyweight increased the CL by 99%. Clearance is reduced in females compared with male patients by 6%
Yamakawa et al. 2011	8.7 (5.3)	2.06 (3)	430 (9.9)		CL–60.2 Vd–67.6 Ka–86.4	PE–63.2	Patients with SLCO1B3 334GG genotype had higher imatinib clearance (36%) than those with TT and TG genotypes

*CL* Clearance (Liter/hr), *Vd *Volume of distribution (Liter), *Ka *Absorption rate constant (per hour), *D1 *Duration for absorption (hour), *IIV *Interindividual variability, *RUV *Residual unexplained variability, *PE *Proportional error, *AE *Additive error, *%RSE *Relative Standard Error

Above table displays the pharmacokinetic parameters and inferences of available/collected studies

### Clinical data

From a total of 49 subjects, six were dropped from the analysis because of the unusually high-drug concentration found during the elimination phase in the data, which did not make any pharmacokinetics sense. External validation was performed using the remaining data from 43 adult CML patients with 145 imatinib plasma concentrations. Blood samples were collected between 0.5 and 24 h after administration of imatinib doses, based on the convenience of patients when they were able to provide samples. The distribution of sample collection time after dosing is depicted in Supplementary Fig. 1. All imatinib concentrations were measured accurately. Most of the patients were in chronic phase (74.4%) of CML. Table 2 displays the clinical and demographic characteristics of the external validation dataset. Imatinib was given orally, with doses ranging from 400 to 800 mg, at a median dose of 400 mg once daily. Samples taken during and after 20 h of administration have been regarded as representing the trough concentration. 43 of the 145 imatinib plasma concentrations were trough concentrations. Only 17 (39.5%) imatinib trough concentrations fell within the therapeutic range of 1.0–1.5 µg/mL [8]; the remaining samples were either above or below this level.

**Table 2:** Patient Clinical and Demographic characteristics (*N* = 43)

Baseline characteristics	Mean ± SD or *N*	Range or percentage
Age (in years)	41.16 ± 10.98	18–68
ECOG.PS Grade 0 Grade 1	25 18	58.1% 41.8%
Gender Male Female	35 8	81.3% 18.7%
Height (cm)	163.8 ± 6.86	148–177.2
Weight (kg)	63.88 ± 10.09	45–86.4
BSA	1.688 ± 0.15	1.3–2.0
Imatinib Trough Concentration (µg/mL) (median) 400 mg 600 mg 800 mg	1.19 1.45 NA	0.71–2.6 0.85–2.58
Biochemistry		
Haemoglobin (g/dL)	12.6 ± 1.5	9.0–16.1
Total count (10^12/L)	4.2 ± 0.9	3.0–8.7
Differential count	5.7 ± 1.9	1.7–11.3
Neutrophils (%)	53.97 ± 10.42	28.2–78.6
Lymphocytes (%)	31.29 ± 9.59	15.5–50.6
Monocytes (%)	5.67 ± 6.18	1.9–44.4
Eosinophils (%)	6.68 ± 8.08	0.8–44.5
Basophils (%)	0.54 ± 0.59	0.1–3.7
Platelets (10^9/L)	225 ± 78.19	104–432
Serum Bilirubin (g/dL)	0.57 ± 0.30	0.2–1.7
SGOT (U/L)	32.23 ± 10.58	15–65
SGPT (U/L)	37.86 ± 16.60	12–86
ALP (U/L)	88.54 ± 27.61	46–179
BUN (mg/dL)	20.5 ± 6.4	9–37.3
Albumin (g/dL)	4.03 ± 0.45	3–4.9
Globulin (g/dL)	3.16 ± 0.48	1.8–4.1
Serum Protein (g/dL)	7.15 ± 0.62	5.79–8.70
Serum Creatinine (mg/dL)	1.09 ± 0.20	0.7–1.5
SNPs		
*ABCB1*		
*rs1128503*		
Wild	21	48.8%
Heterozygous	16	37.2%
Homozygous	6	13.9%
*rs2032582*		
Wild	22	51.1%
Heterozygous	15	34.8%
Homozygous	6	13.9%
*rs1045642*		
Wild	23	53.4%
Heterozygous	15	34.8%
Homozygous	5	11.6%
*ABCG2 (rs2231142)*		
Wild	35	81.3%
Heterozygous	8	18.6%
CYP3A4*12 (rs12721629)		
Wild	43	100%
CYP3A4*1B (rs2740574)		
Wild	39	90.6%
Heterozygous	2	4.6%
NA	2	4.6%
CYP3A4*18 (rs8371759)		
Wild	41	95.4%
NA	2	4.6%

### External validation

**Fig. 2:**
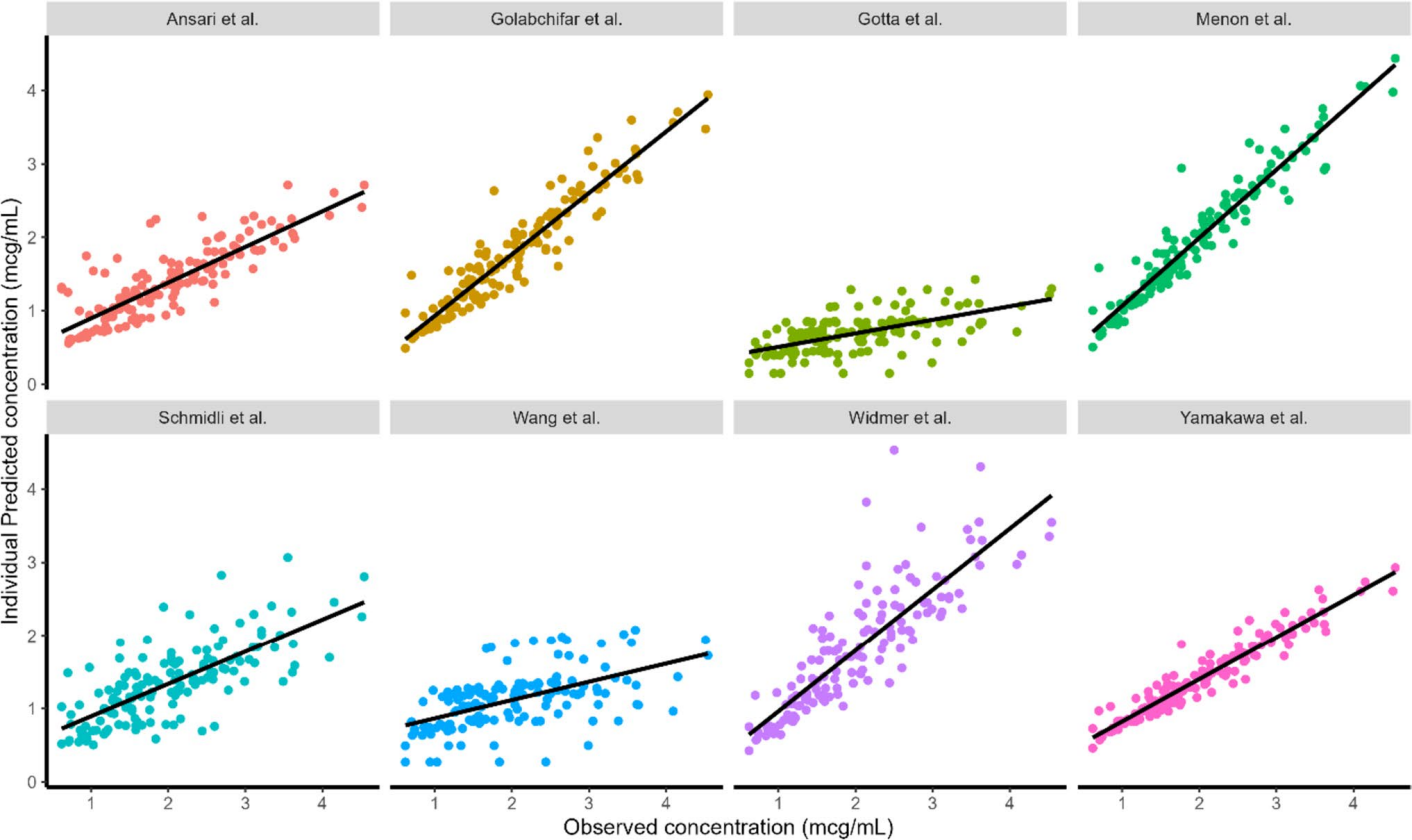
Individual predicted concentrations by each model vs. observed concentrations from the present clinical study

**Fig. 3:**
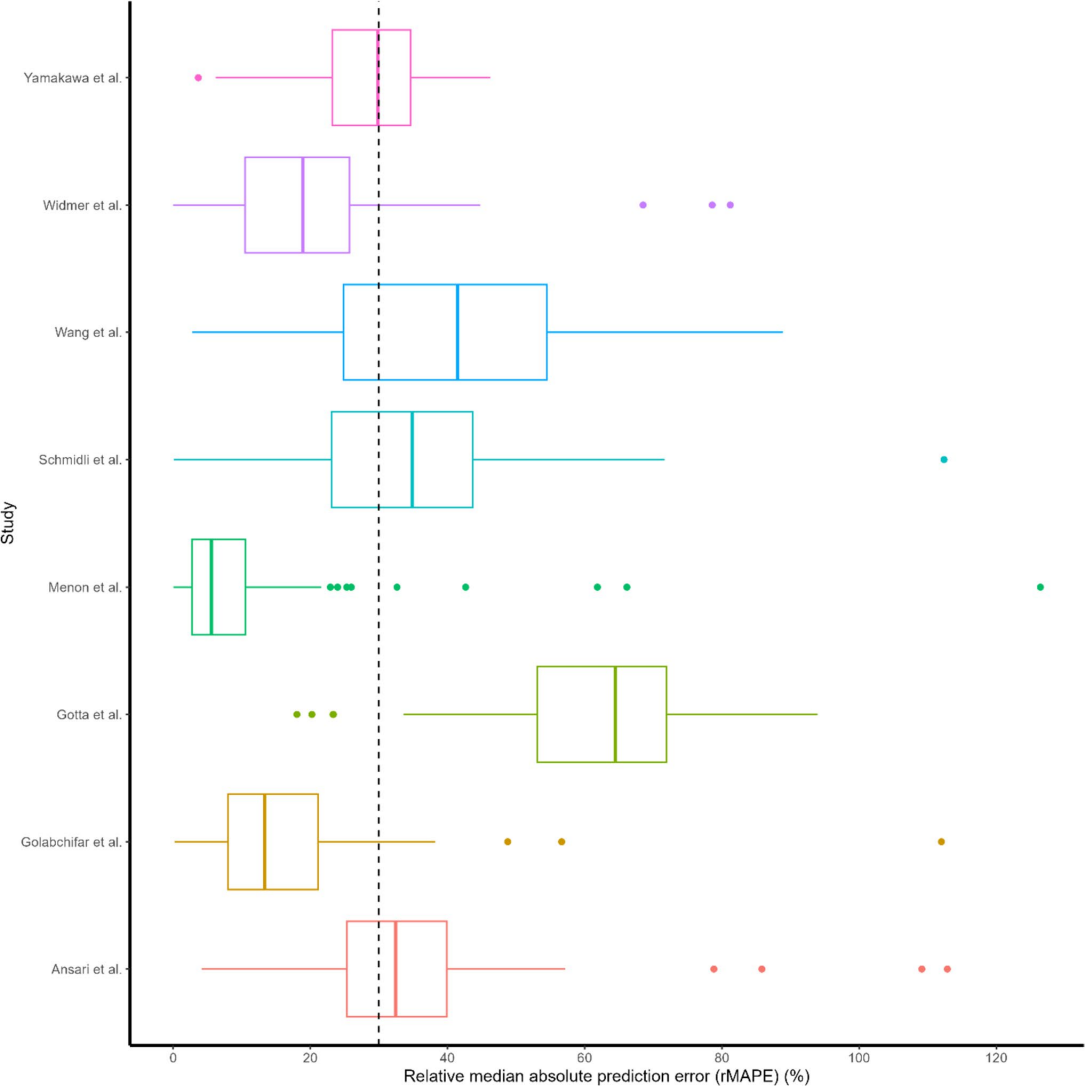
Relative median aboslute prediction error distribution of the imatinib PopPK models evaluated using external clinical dataset. Dashed black line, rMAPE equal to ± 30% (acceptable bias)

The findings of the model performance based on prediction-based diagnostics using the 8 PopPK models selected are shown in Table 3. The plot of observed vs. individual predicted concentrations for each model is depicted in Fig. 2. The PopPK model developed by Golabchifer et al., Menon et al., and Widmer et al. had good prediction ability. Other PopPK models that were tested underpredicted imatinib concentrations in our population. However, the Menon et al. model outperformed the Golabchifer and Widmer model in terms of prediction ability, with rMPE of 0.98% (between 20%), rMAPE of 5.59% (between 30%), and the lowest rRMSE of 0.16 (close to zero). Figure 3 demonstrates the precision of the imatinib PopPK models that were examined [29]. The Menon et al.’s model is a one-compartment with first-order elimination and zero-order absorption. Body weight was included allometrically in the model to explain CL and Vd.

**Table 3:** An overview of prediction-based diagnostics

Author, year	rMPE (%)	rMAPE (%)	rRMSE
Ansari et al. 2016	− 25.89	32.45	0.36
Golabchifar et al. 2014	− 10.46	13.36	0.19
Gota et al. 2014	− 62.18	64.45	0.63
Menon et al. 2008	0.98	5.59	0.16
Schmidli et al. 2004	− 28.42	34.87	0.38
Wang et al. 2018	− 38.62	41.46	0.44
Widmer et al. 2006	− 9.72	18.89	0.23
Yamakawa et al. 2011	− 27.35	29.86	0.29

The table above depicts each model's prediction performance for clinical data based on the rMPE, rMAPE, rRMSE

### Simulation approach for designing dosing nomogram

The Menon et al. model was used to develop the dosing nomogram in the weight range of 45–120 kg (Table 4). Virtual subjects were first created for the range of covariates with narrow intervals to create a dosing nomogram. Body weights ranging from 40 to 120 kg were divided into groups with 3 kg intervals. The PK profile for different weight groups was simulated with 1000 subjects in each group using imatinib doses ranging from 400 to 800 mg with a 100 mg interval. All weight groups were tested with each dosage regimen to determine the best-dosage regimen for patients. The target trough concentration for the simulation was set at 1.0–1.5 µg/mL, which falls within the acceptable therapeutic range. More than 95% of the simulations in each weight group fell within the therapeutic range. The clinician may use the recommended dosing nomogram to maintain the imatinib concentration in the therapeutic range.

**Table 4:** Dosing recommendation for CML patients based on body weight

Weight range (kg)	Imatinib dose
45–57	400 mg
58–84	500 mg
85–108	600 mg
109–120	700 mg

400 mg is the standard dose for chronic phase and 600 mg is the standard dose for accelerated and blast phase

Simulations for these recommendations were carried out using final selected model for the weight from the range 45–120 kg. This weight range was taken from the Menon et al. article. These recommendations are expected to result in a trough concentration between 1.0 and 1.5 µg/mL

## Discussion

In the last decade, a number of population pharmacokinetic studies were published about many drugs in different populations. Predictability and applicability of these models outside their study settings have not been evaluated. There is a need for evaluating these models using either external data from other published studies or from prospective clinical studies. Such evaluations will lend these models amenable for application in clinical situation that needs precision dosing [16, 30]. All the chosen PopPK models were externally evaluated with our clinical data. In these evaluations, the model reported by Golabchifar et al. [23], Menon et al. [29], and Widmer et al. [27] performed well with the acceptable rMPE (%), rMAPE, and rRMSE values. Using rMPE (%), rMAPE, and rRMSE as a model evaluation metrics will assess both bias and precision [31]. The model proposed by Menon et al. was chosen as the final model for dosing nomogram development because it had the lowest rMPE, rMAPE, and rRMSE. All the other models underpredicted the concentrations, and their evaluation metrics were unsatisfactory for the dosing recommendations, this could be due to the differences in the demographics, biochemical parameters and the ethnicity of the study population. One possible explanation for the better performance of Menon et al.’s PopPK model could be the inclusion of weight as a covariate on CL and Vd. In contrast, Golabchifar et al.’s model included no covariates on the PK parameters, whereas Widmer et al.’s model included age, gender, stage of diagnosis, and weight.The clinical dataset available for external evaluation had 49 patients’ data, but 6 patients were dropped from the study because of unusually high-drug concentrations in those subjects which did not make any pharmacokinetics sense. These concentrations were observed between 9 and 10 h and were three to four times higher than the typical mean concentration of 2.04 µg/mL observed during that time window. Imatinib has a high inter-individual variability in the pharmacokinetic parameters related to the processes of absorption, distribution, metabolism and elimination. This inter-individual variability is purportedly due to factors such as genetic polymorphisms in transporters and metabolizing enzymes, body weight, age, gender, white blood cell (WBCs) count and hemoglobin (Hb) value. Patients with higher body-weight and Hb values reported to have increased CL by 12% and 86% and increase in the Vd by 32% and 60% respectively. If a patient had reduced WBC count by the factor of two, then both CL and Vd increased by 8% and 5% respectively [25]. Takahashi et al. stated that therapeutic drug monitoring along with ABCG2 421 C > A genotyping might be helpful in improved imatinib therapy management as patients with ABCG2 421C/A or A/A genotype were associated with a higher trough concentration than ABCG2 421C/C genotype [14] but when we tried to predict with ABCG2 as a covariate we found no significant impact on the trough concentration among these genotypes. Nevertheless, therapeutic drug monitoring might be important to understand whether the patient’s trough concentrations is above or below the required threshold value (1–1.5 µg/mL).

Several studies have reported that the trough concentration for imatinib is higher in patients with major molecular response (MMR) than in patients without MMR. The same pattern was recognized for complete cytogenetic response (CCyR). The patients had significantly higher MMR (minimum of 3 log reduction in the level of BCR-ABL transcript from a standard baseline after initiation of therapy), when the trough concentration was found to be higher than 1 µg/mL [13–15, 33]. There is still a debate on the therapeutic range of imatinib in CML therapy [34]. Main reason attributed to the treatment resistance for imatinib in CML patients was trough concentrations lower than 1 µg/mL [35]. Higher imatinib trough concentrations of 1.5 µg/mL & 3 µg/mL have been reported to be associated with toxicities such as neutropenia, grade 3 myalgia, fluid retention and eyelid edema [5, 9, 10].

In Tyrosine Kinase Inhibitor Optimization and Selectivity (TOPS) trial, it has been reported that there is an association between the imatinib C_trough_ and the frequency of AEs. Imatinib C_trough_ above 1.165 µg/mL had high MMR and CCyR but C_trough_ above 3.18 µg/mL has resulted in higher frequency of grade 3 or 4 AEs. However, the upper limit for the therapeutic window have not been reported [36]. Recent studies have suggested that the imatinib C_trough_ can be maintained between 1 and 1.5 µg/mL because of higher probability of therapeutic success without drug resistance and lower probability of developing AEs [7, 8, 37]. Based on these reports, we considered the window between 1 and 1.5 µg/mL for proposing dosage regimen for various body-weight ranges.

Pharmacometric simulations using the identified generalizable model for the weight range of 45–120 kg with standard regimen resulted in C_trough_ values less than 1 µg/mL in certain weight groups. In certain body-weight groups, trough concentrations were well over 1.5 µg/mL. These simulations showed the need for varied doses in different body-weight groups as opposed to the present fixed dosage regimen to all the body-weight ranges to attain the therapeutic range.

The dosage regimen proposed is based on the simulation for the typical subjects with specific body weight considering variability. The current recommendation remains a broad guideline for initial doses in patients with specific body weights and the doses may have to be adjusted again based on the trough levels of imatinib, if they are outside the desired ranges.

This study has several limitations, including a small number of patients (*n* = 43) and imatinib plasma samples (*n* = 145), which may limit the robustness of the results, differences between structural and stochastic PopPK models due to the high BSV in imatinib PK previously described in the literature, and the impact of ethnicity on model performance. The findings of this study also suggest that additional imatinib TDM samples are required when the patient did not show signs of improvement in MMR and CCyR.

## Conclusion

Systematic review was performed to identify all the PopPK models of imatinib. All the selected models were externally evaluated using the clinical data from the current study setting. The PopPK model reported by Menon et al. met all the qualification requirements, with an rMPE of 0.98%, rMAPE of 5.59% and rRMSE of 0.16 and was thus identified as a model with better prediction capability. The Menon et al.’s model is a one-compartment model with zero-order absorption where body weight is allometrically scaled on clearance and volume of distribution. Based on this PopPK model, a dosing regimen was proposed for various weight ranges of CML patients. This current dosing regimen is expected to aid clinicians in determining the best-dosage regimen for maintaining imatinib trough concentrations within the optimal range.

### Supplementary Information

Below is the link to the electronic supplementary material.

Supplementary file1 (DOCX 26 KB)

Supplementary file2 (DOCX 15 KB)

Supplementary file3 (DOCX 13 KB)

## Data Availability

Not Applicable.
