# Supplementary material for: Predictive performance of population pharmacokinetic models of imatinib in chronic myeloid leukemia patients
Source: Cancer Chemother Pharmacol. 2024 Mar 5;94(1):35–44. doi: 10.1007/s00280-024-04644-w (PMC11258086; doi:10.1007/s00280-024-04644-w)
Supplement: Supplementary file 1 — Supplementary file1 (DOCX 26 KB) [file 280_2024_4644_MOESM1_ESM.docx]

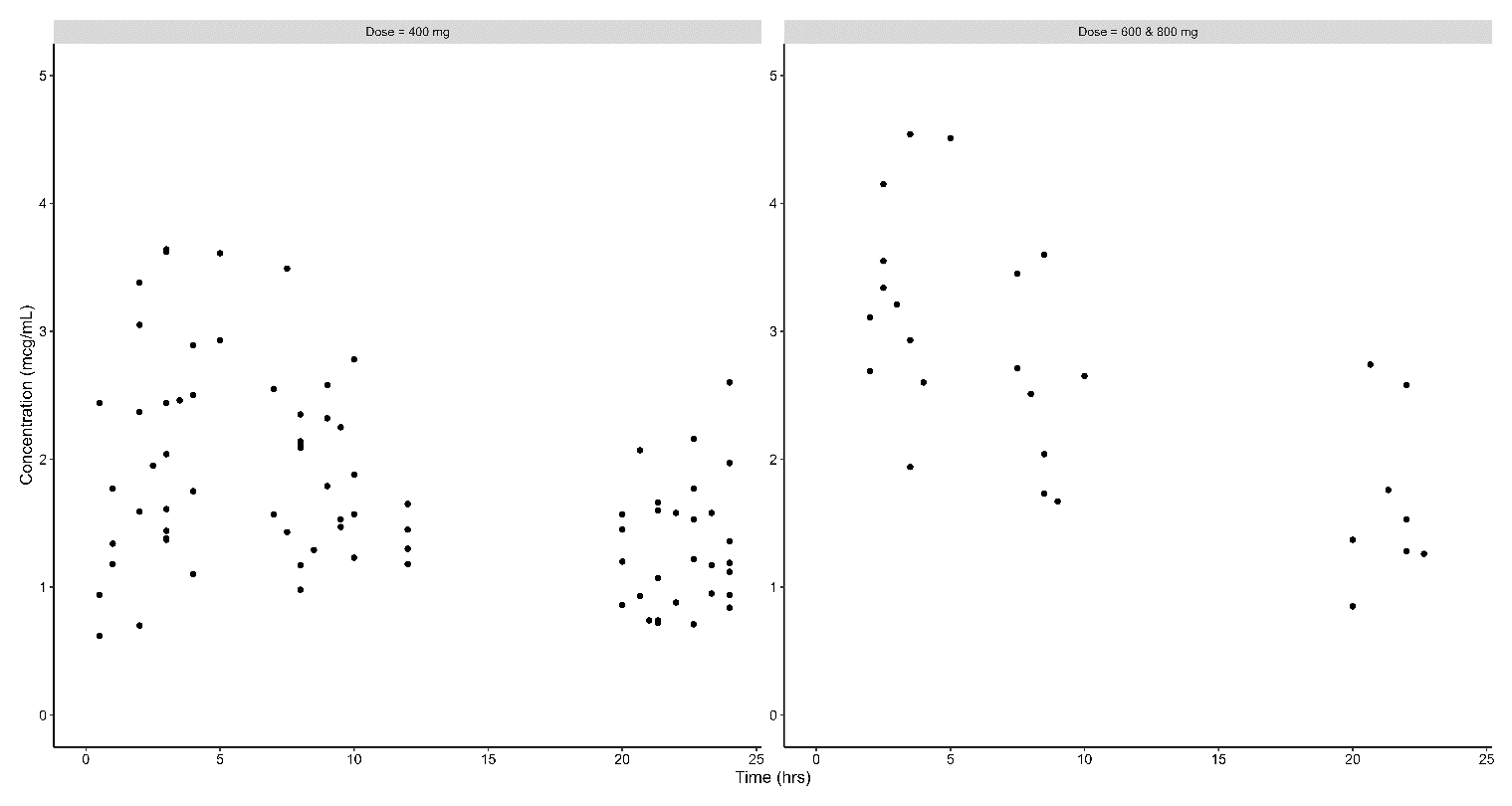
**Supplementary Figure 1. Time vs Concentration plot based on dose for the clinical data**

*Since there was only one patient receiving the 800 mg dose, that patient has been combined into the 600 mg dose plot.
