# Supplementary material for: Predictive performance of population pharmacokinetic models of imatinib in chronic myeloid leukemia patients
Source: Cancer Chemother Pharmacol. 2024 Mar 5;94(1):35–44. doi: 10.1007/s00280-024-04644-w (PMC11258086; doi:10.1007/s00280-024-04644-w)
Supplement: Supplementary file 2 — Supplementary file2 (DOCX 15 KB) [file 280_2024_4644_MOESM2_ESM.docx]

**Supplementary Table 1: Study details and demographic characteristics of reported studies**

| **Author, Year** | **Study population** | | **Dose**  **(mg)** | **Weight**  **(Kgs)** | **One compartment model (absorption type)** | **Covariates analysed** |
| --- | --- | --- | --- | --- | --- | --- |
| Ansari et al, 2016 | Iran | 400 | | 40-100 | First order | Weight |
| Golabchifar et al, 2014 | Iran | 300-800 | | 47-105 | Zero order | NA |
| Gota et al, 2014 | Europe | 100-1200 | | NA | Zero order | Age, Gender |
| Menon et al, 2008 | USA | 260-570 | | 40.3-119.2 | Zero order | Weight |
| Schmidli et al, 2004 | USA | 400 | | 40-169.5 | Zero order | Weight, Hb, WBC, OCC |
| Wang et al, 2018 | China | 400 | | 42.5-101 | First order | Age, gender, ethnicity, stage of disease, duration of treatment, genotypes |
| Widmer et al, 2006 | Switzerland | 400 | | 44-110 | First order | Weight, gender, age and stage of diagnosis |
| Yamakawa et al, 2011 | Japan | 100-600 | | 40-83 | First order | Genotypes |

* Hb-Hemoglobin, WBC-White blood cell, OCC-Occasion

* This table represents the demographic characteristics and study details of the population pharmacokinetic articles selected through the systematic review.
