# Supplementary material for: Predictive performance of population pharmacokinetic models of imatinib in chronic myeloid leukemia patients
Source: Cancer Chemother Pharmacol. 2024 Mar 5;94(1):35–44. doi: 10.1007/s00280-024-04644-w (PMC11258086; doi:10.1007/s00280-024-04644-w)
Supplement: Supplementary file 3 — Supplementary file3 (DOCX 13 KB) [file 280_2024_4644_MOESM3_ESM.docx]

**Supplementary Table 2: Search Strategy**

| **Data Base** | **Search Strategy** | **No. of Articles** |
| --- | --- | --- |
| PubMed | ((“IMATINIB”) AND ((“POPULATION PHARMACOKINETIC*”) OR (“POPPK”))) AND ((“CHRONIC MYELOID LEUKEMIA*”) OR (“CML”)) | 13 |
| Web of Science | ((“IMATINIB”) AND ((“POPULATION PHARMACOKINETIC*”) OR (“POPPK”))) AND ((“CHRONIC MYELOID LEUKEMIA*”) OR (“CML”)) | 65 |
